# P320: Epidemiological study drug administration routes (DAR) in the department of pediatrics Gabriel Touré hospital, Mali

**DOI:** 10.1186/2047-2994-2-S1-P320

**Published:** 2013-06-20

**Authors:** DK Minta, M Sylla, A Camara, AM Traoré, M Diakité, H Cissé, L Bengaly, M Sacko, B Traoré, AS Kaya, AT Sidibé, HA Traoré, T Sidibé, MM Keita

**Affiliations:** 1 Ministère de la santé , Hôpital Gabriel Touré, Bamako, Mali; 2 Service de Pédiaterie, CHU Gabriel Touré, Bamako, Mali; 3 Services de Maladies infectieuses, CHU du Point G, Bamako, Mali; 4 Service de Pharmacie Hospitalière, CHU Gabriel Touré, Bamako, Mali; 5 Département de Santé publique, Faculté de Médecine, Université de Bamako, Bamako, Mali; 6 Servide de Pédiaterie, CHU du Point G, Bamako, Mali; 7 Service de Médecine interne, CHU du Point G, Bamako, Mali

## Introduction

Injectable route seems to be the most frequently used in health facilities in resource-limited.

## Objectives

In order to better understand this issue still under documented, we conducted a longitudinal study of DAR use in the general pediatric ward of the Gabriel Touré Hospital to Bamako during 6 months.

## Methods

We are interested in the routes of administration applied to a population of 300 children with a sex ratio (M / F) = 1.3. Their average age was 2 years ± 1.

## Results

Assumptions diagnostic from the admission to discharge patients underwent a change both their frequency and their formulation as well malaria (37.4% vs 39.7% at 72 hours), pneumonia (19% vs 20% at 72 hours), and nephrotic syndrome (2.2% vs. 5.1% in 72 hours) occurred most commonly mentioned.

Treatments were prescribed according to the diagnostic were administered per injection route at admission (76.6%) to72th hours (70%) and the output (36.3%). Complications were noted inflammation of the catheter puncture sites (21.8%) at admission, 18% after 5 days of hospitalization, the abscess puncture MI (2.1%). The mean duration of hospitalization recorded was 7.6 ± 3.7 days and lethality was 11%.

## Conclusion

Injection is the most widely used in the pediatric unit III in connection with the most common pathologies. A detailed study would be needed to assess the adequacy of the diagnostic hypotheses and routes of administration. The injection volume exposed to the risk of Accidental exposure to blood staff and patients.

## Disclosure of interest

None declared

